# Paraneoplastic Cerebellar Degeneration With P/Q-VGCC vs Yo Autoantibodies

**DOI:** 10.1212/NXI.0000000000200006

**Published:** 2022-05-31

**Authors:** Michael Winklehner, Jan Bauer, Verena Endmayr, Carmen Schwaiger, Gerda Ricken, Masakatsu Motomura, Shunsuke Yoshimura, Hiroshi Shintaku, Kinya Ishikawa, Yukio Tsuura, Takahiro Iizuka, Takanori Yokota, Takashi Irioka, Romana Höftberger

**Affiliations:** From the Division of Neuropathology and Neurochemistry (M.W., V.E., C.S., G.R., R.H.), Department of Neurology, and Department of Neuroimmunology (J.B.), Center for Brain Research, Medical University of Vienna, Austria; Department of Electrical and Electronics Engineering (M.M.), Faculty of Engineering, Nagasaki Institute of Applied Science; Department of Neurology and Strokology (S.Y.), Nagasaki University Hospital; Neurology Clinic with Neuromorphomics Laboratory (H.S.), Nitobe Memorial Nakano General Hospital, Tokyo; Division of Surgical Pathology (H.S.), Tokyo Medical and Dental University Hospital; The Center for Personalized Medicine for Healthy Aging (K.I.), Tokyo Medical and Dental University; Departments of Diagnostic Pathology and Clinical Laboratory (Y.T.), Yokosuka Kyosai Hospital, Kanagawa; Department of Neurology (T. Iizuka), Kitasato University School of Medicine, Kanagawa; Department of Neurology and Neurological Science (T.Y.), Graduate School, Tokyo Medical and Dental University; and Department of Neurology (T. Irioka), Yokosuka Kyosai Hospital, Kanagawa, Japan.

## Abstract

**Background and Objectives:**

Paraneoplastic cerebellar degeneration (PCD) is characterized by a widespread loss of Purkinje cells (PCs) and may be associated with autoantibodies against intracellular antigens such as Yo or cell surface neuronal antigens such as the P/Q-type voltage-gated calcium channel (P/Q-VGCC). Although the intracellular location of the target antigen in anti–Yo-PCD supports a T cell–mediated pathology, the immune mechanisms in anti–P/Q-VGCC-PCD remain unclear. In this study, we compare neuropathologic characteristics of PCD with anti–P/Q-VGCC and anti-Yo autoantibodies in an archival autopsy cohort.

**Methods:**

We performed neuropathology, immunohistochemistry, and multiplex immunofluorescence on formalin-fixed and paraffin-embedded brain tissue of 1 anti–P/Q-VGCC, 2 anti–Yo-PCD autopsy cases and controls.

**Results:**

Anti–Yo-PCD revealed a diffuse and widespread PC loss together with microglial nodules with pSTAT1^+^ and CD8^+^granzymeB^+^ T cells and neuronal upregulation of major histocompatibility complex (MHC) Class I molecules. Some neurons showed a cytoplasmic immunoglobulin G (IgG) staining. In contrast, PC loss in anti–P/Q-VGCC-PCD was focal and predominantly affected the upper vermis, whereas caudal regions and lateral hemispheres were spared. Inflammation was characterized by scattered CD8^+^ T cells, single CD20^+^/CD79a^+^ B/plasma cells, and an IgG staining of the neuropil in the molecular layer of the cerebellar cortex and neuronal cytoplasms. No complement deposition or MHC-I upregulation was detected. Moreover, synaptophysin was reduced, and neuronal P/Q-VGCC was downregulated. In affected areas, axonal spheroids and the accumulation of amyloid precursor protein and glucose-regulated protein 78 in PCs indicate endoplasmatic reticulum stress and impairment of axonal transport. In both PCD types, calbindin expression was reduced or lost in the remaining PCs.

**Discussion:**

Anti–Yo-PCD showed characteristic features of a T cell–mediated pathology, whereas this was not observed in 1 case of anti–P/Q-VGCC-PCD. Our findings support a pathogenic role of anti–P/Q-VGCC autoantibodies in causing neuronal dysfunction, probably due to altered synaptic transmission resulting in calcium dysregulation and subsequent PC death. Because disease progression may lead to irreversible PC loss, anti–P/Q-VGCC-PCD patients could benefit from early oncologic and immunologic therapies.

Paraneoplastic cerebellar degeneration (PCD) is characterized by an extensive loss of Purkinje cells (PCs) and frequently associated with inflammatory infiltrates.^1,2^ Clinically, patients develop a rapidly progressive cerebellar syndrome commonly suffering from gait instability, vertigo, ataxia, dysarthria or ocular motor abnormalities and are associated with specific types of cancer.^3,4^ Autoantibodies can be directed against intracellular or cell surface epitopes, and underlying immunopathogenic and neurotoxic mechanisms might therefore be fundamentally different.^5^

In about 60% of PCD cases, high-risk antibodies against intracellular antigens like Yo (CDR2/CDR2L) or Tr (DNER) are present.^4,5^ Anti-Yo antibodies are frequently associated with ovarian or breast cancer.^4,6^ Neuropathologic studies of anti–Yo-PCD showed typical, nodular inflammatory infiltrates within the PC layer comprising cytotoxic T cells (CTLs) and activated microglia.^3,7^ Moreover, preclinical studies and experimental data demonstrating major histocompatibility complex (MHC) Class I–restricted CDR2-specific CTLs support the perception of a T cell–mediated pathogenesis.^6,8,9^ In addition, in vitro studies demonstrated an internalization of anti-Yo antibodies resulting in an alteration of calcium homeostasis of PCs, suggesting a pathogenic antibody role possibly by interfering with ribosome function via binding to CDR2L.^10-12^

P/Q-type voltage-gated calcium channel (P/Q-VGCC) antibodies are prevalent in about 40% of patients with PCD with small-cell lung cancer.^13,14^ The antibodies are commonly associated with Lambert-Eaton myasthenic syndrome (LEMS) and may additionally present with PCD (with or without LEMS), possibly due to the recognition of varying target epitopes.^3,13,15^ At the neuromuscular junction, P/Q-VGCC antibodies were shown to cause a downregulation and blocking of Ca_v_ 2.1 receptors.^16,17^ In PCD with anti–P/Q-VGCC antibodies, the pathogenic mechanisms are unclear. Autopsies of patients with PCD-LEMS with anti–P/Q-VGCC antibodies are rare and mainly focused on the expression level of P/Q-VGCC, showing a significant reduction particularly within the molecular layer of the cerebellar cortex.^18^ Antibodies against the major immunogenic region of P/Q-VGCC were shown to alter cerebellar synaptic transmission by inhibiting neuronal P/Q-VGCCs.^15^ In mice, intrathecal injection of anti–P/Q-VGCC antibodies from 1 patient with PCD-LEMS caused reversible cerebellar symptoms.^19^ Patients' immunoglobulin G (IgG) also caused neuronal antibody internalization and subsequent PC death in a rat cerebellar slice culture.^20^ High antibody prevalence in patients with PCD, partial response to antibody-depleting therapies, and intrathecal antibody production in a subset of patients furthermore suggest anti–P/Q-VGCC antibodies to be pathogenic in PCD.^2,3,13,18,20^ Nevertheless, pathomechanisms that result in disease progression and unsuccessful immunotherapy are still unresolved.^13,21^

We present human autopsy data comparing in-depth neuropathologic characteristics of patients with PCD with anti–P/Q-VGCC and anti-Yo autoantibodies. Our results support the concept of an antibody-mediated disease in anti–P/Q-VGCC-PCD.

## Methods

### Sample Characterization

The study was performed using an archival collection of autopsies from 3 patients with PCD, 2 with anti-Yo and 1 with anti–P/Q-VGCC antibodies, and 2 controls. The cases were collected from 1995 to 2018 and archived at the Division of Neuropathology and Neurochemistry, Department of Neurology, Medical University of Vienna, Austria (4 autopsies) and the Departments of Diagnostic Pathology and Clinical Laboratory, Yokosuka Kyosai Hospital, Japan (1 autopsy).

### Neuropathology and Immunohistochemistry

Neuropathologic analysis and immunohistochemistry (IHC) were performed on small cerebellar sections from formalin-fixed and paraffin-embedded (FFPE) tissue blocks. Hematoxylin and eosin and a set of primary antibodies, which are summarized in eTable 1 (links.lww.com/NXI/A726), were used to stain the sections. For double immunolabeling using primary antibodies derived from different species, the same antigen retrieval techniques were applied (eTable 1). Immunoreactivity was subsequently visualized by using alkaline phosphatase–conjugated secondary antibodies for subsequent development with Fast Blue BB salt as well as biotinylated secondary antibodies and peroxidase-conjugated streptavidin for subsequent development with aminoethyl carbazole.^22^ Slide scanning was performed on a NanoZoomer 2.0-HT digital slide scanner C9600 (Hamamatsu Photonics, Hamamatsu, Japan).

### Multiplex Immunofluorescence

Multiplex immunofluorescence was performed on small cerebellar sections from FFPE tissue blocks with OPAL reagents from PerkinElmer as described in the PerkinElmer Multiplex IHC manual. Primary antibodies used are summarized in eTable 1 (links.lww.com/NXI/A726, method: IF). Pretreatment (heating) in a Braun household vegetable cooking device was performed in AR9 antigen retrieval buffer from PerkinElmer for 40 minutes before the first antibody and with AR6 antigen retrieval buffer from PerkinElmer for 30 minutes between each antibody staining.

### Standard Protocol Approvals, Registrations, and Patient Consents

The study was approved by the Institutional Review Board of the Medical University of Vienna (EK 1123/2015 and 1636/2019).

### Data Availability

Data can be made available from the corresponding author on reasonable request and after approval from the ethics review board at the Medical University of Vienna.

## Results

We studied postmortem brain tissue of 3 patients with PCD (1 woman, 58–69 years old), 2 with anti-Yo and 1 with cell surface anti–P/Q-VGCC autoantibodies, and controls. Clinical data were obtained from treating physicians.

### Case Reports

#### Anti–Yo-PCD

Detailed clinical information of the first patient was previously published.^7^ Briefly, a 66-year-old male patient with the diagnosis of an invasive non–small-cell lung cancer presented with progressive ataxia, vertigo, and speech disturbances. Following the detection of anti-Yo antibodies using indirect immunofluorescence and immunoblot, chemotherapy was initiated. However, general and neurologic conditions deteriorated, and the patient died 5 months after initial neurologic symptoms due to pneumonia. The second patient was a 58-year-old woman presenting with a progressive unstable gait and change in motor speech. MRI revealed no cranial or spinal abnormalities. CSF analysis was unremarkable. Electroencephalography was normal. Additional electrophysiologic examination showed a mildly delayed nerve conduction and decreased motoric amplitude, indicating possible neuropathies of the lower extremities. Initial therapy with 500 mg methylprednisolone for 3 days and 600 mg alpha lipoic acid followed by a stay at the rehabilitation unit did not achieve improvement. Cerebellar symptoms progressed to a hyporeflexia and areflexia in the upper and lower extremities, respectively, a latent paresis of lower limbs, dysarthria, and upper limb intention tremor (modified Rankin Scale [mRS] score 4). Subsequent autoantibody screening was positive for anti-Yo antibodies. Analysis of relevant tumor markers revealed elevated levels of CA-125, suggestive for ovarian carcinoma. However, CT, MRI, and sonography did not reveal a tumor. The patient did not respond to repeated therapy cycles with steroids and developed vertical nystagmus, severe dysarthria, incomprehensible speech, involuntary movements, weak motor reflexes, and areflexia of the upper and lower extremities, respectively. Following sepsis, anemia, and hypokalemia, the patient deceased 3 months after her first neurologic admission. At autopsy, no tumor was found macroscopically, and the brainstem and cerebellum were processed for further histopathologic investigation.

#### Anti–P/Q-VGCC-PCD

**Figure 1:**
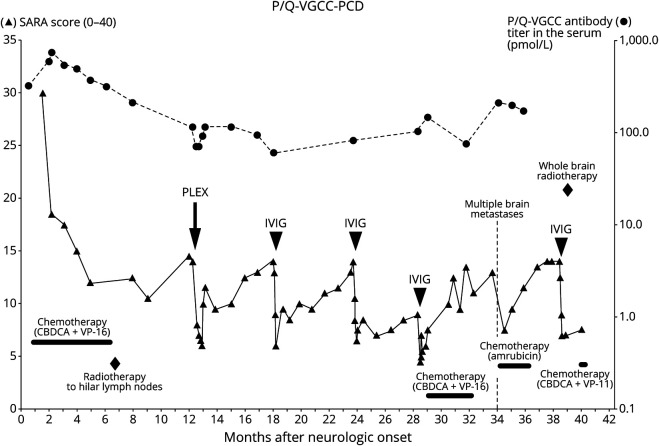
Clinical Course of Anti–P/Q-VGCC-PCD The clinical course shows a 42-month disease duration and positive effects of oncologic and immunologic therapies on cerebellar symptoms. (▲) Left y-axis: the SARA score on cerebellar symptoms ranges from 0 (asymptomatic) to 40. (●) Right y-axis: anti–P/Q-VGCC antibody titers in the serum (pmol/L). CBCDA = carboplatin; CPT-11 = irinotecan; IVIG = IV immunoglobulin; PC = Purkinje cell; PCD = paraneoplastic cerebellar degeneration; PLEX = plasma exchange; P/Q-VGCC = P/Q-type voltage-gated calcium channel; SARA = the Scale for the Assessment and Rating of Ataxia; VP-16 = etoposide.

A 69-year-old male patient initially developed vertigo, dysarthria, and gait instability. Three weeks later, he was having oscillopsia and an inability to sit without assistance (mRS score 5). Neurologic examination at admission revealed dysarthria with explosive speech, truncal and limb ataxia, and a downbeat nystagmus. No motor weakness, sensory loss, or autonomic dysfunction was present. CSF examination showed a mild lymphocytosis (15 cells/μL) and an elevated protein concentration (52 mg/dL) without oligoclonal bands. Brain MRI and electrophysiologic examination revealed no abnormalities, particularly no signs for LEMS. Thoracic CT and transbronchial biopsy led to the diagnosis of a small-cell lung cancer. Extensive screening for autoantibodies was negative for intracellular antibodies (Hu, Ri, Yo, PCA-2, Tr, ANNA-3, AGNA-1, amphiphysin, CRMP-5, and GAD) and surface antibodies (NMDAR, AMPAR, GABA(A)R, GABA(B)R, mGluR1, mGluR5, LGI1, Caspr2, DPPX, neurexin-3α, and IgLON5). However, radioimmunoprecipitation assays revealed anti–P/Q-VGCC autoantibodies in the serum (319 pmol/L, normal range <20 pmol/L).^23^ Details on the clinical course are shown in Figure 1. After initiating chemotherapy, the patient improved significantly and was able to walk independently after 1 month (mRS score 2). Cycles of subsequent immunotherapies including plasma exchange (PLEX) and IV immunoglobulins (IVIGs) administrations (0.4 g/kg for 5 days) showed quick and solid responses, although they were always followed by a slowly progressive clinical deterioration. Nevertheless, after the detection of multiple asymptomatic brain metastases, the patient subsequently developed paraparesis and urinary retention and deceased 42 months after disease onset. Autopsy revealed metastases in subependymal regions of the lateral, third, and fourth ventricles, in lumbar dorsal root ganglia, lumbosacral nerve roots, and in the leptomeninges of the cerebellum, medulla, and spinal cord. The left cerebellar hemisphere was macroscopically unremarkable without signs of atrophy and used for histopathologic examinations.

### Neuropathology

#### Pattern of Purkinje and Golgi Cell Loss in Anti–P/Q-VGCC-PCD vs Anti–Yo-PCD

**Figure 2:**
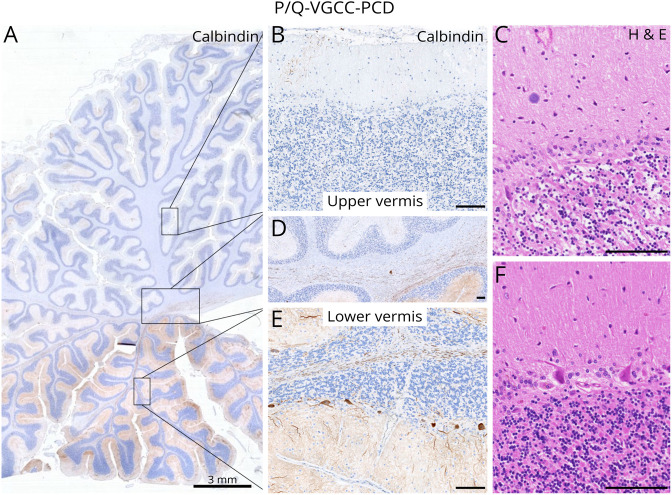
Focal Neuronal Loss in the Vermis in Anti–P/Q-VGCC-PCD Topographic distribution of PC loss shows a focal affection of the upper vermis associated with Bergmann astrogliosis (A–D) and the remaining PCs within the lower vermis (E and F). Scale bars: 100 μm (except for A: 3 mm). PC = Purkinje cell; PCD = paraneoplastic cerebellar degeneration; P/Q-VGCC = P/Q-type voltage-gated calcium channel.

**Figure 3:**
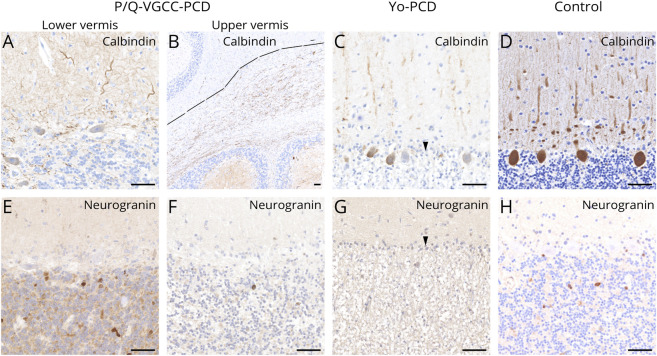
Pattern of Purkinje and Golgi Cell Loss in PCD Subtypes Single remaining PCs in PCD subtypes showed weak or no calbindin immunoreactivity (A–C) and were accompanied by Bergmann astrogliosis (C and G, arrowheads), compared with controls (D). PCs and calbindin expression were lost in the upper vermis in anti–P/Q-VGCC-PCD (B, line marks transition zone from lower to upper vermis). Neurogranin-marked Golgi cells were present in the granular cell layer of the lower vermis in anti–P/Q-VGCC-PCD (E) and in controls (H), coextensively reduced in the upper vermis in anti–P/Q-VGCC-PCD (F), and lost in anti–Yo-PCD (G). Scale bars: 50 μm. PC = Purkinje cell; PCD = paraneoplastic cerebellar degeneration; P/Q-VGCC = P/Q-type voltage-gated calcium channel.

In anti–P/Q-VGCC-PCD, the loss of PCs predominantly affected the upper vermis (Figure 2, A–D), whereas caudal regions and lateral hemispheres were spared (Figures 2, E and F and 3A). In both PCD subtypes, calbindin was lost or reduced in the remaining PCs, compared with controls (Figure 3, A–D). In anti–Yo-PCD, PC loss was almost complete (Figure 3, C and G, arrowheads) and associated with Bergmann astrogliosis. In addition, we found a severe loss of neurons within the dentate nucleus (data not shown). In areas with PC loss, neurogranin-labeled Golgi cells were coextensively reduced in both PCD subtypes, compared with controls (Figure 3, E–H).

#### Characterization of Inflammation in Anti–Yo-PCD vs Anti–P/Q-VGCC-PCD

**Figure 4:**
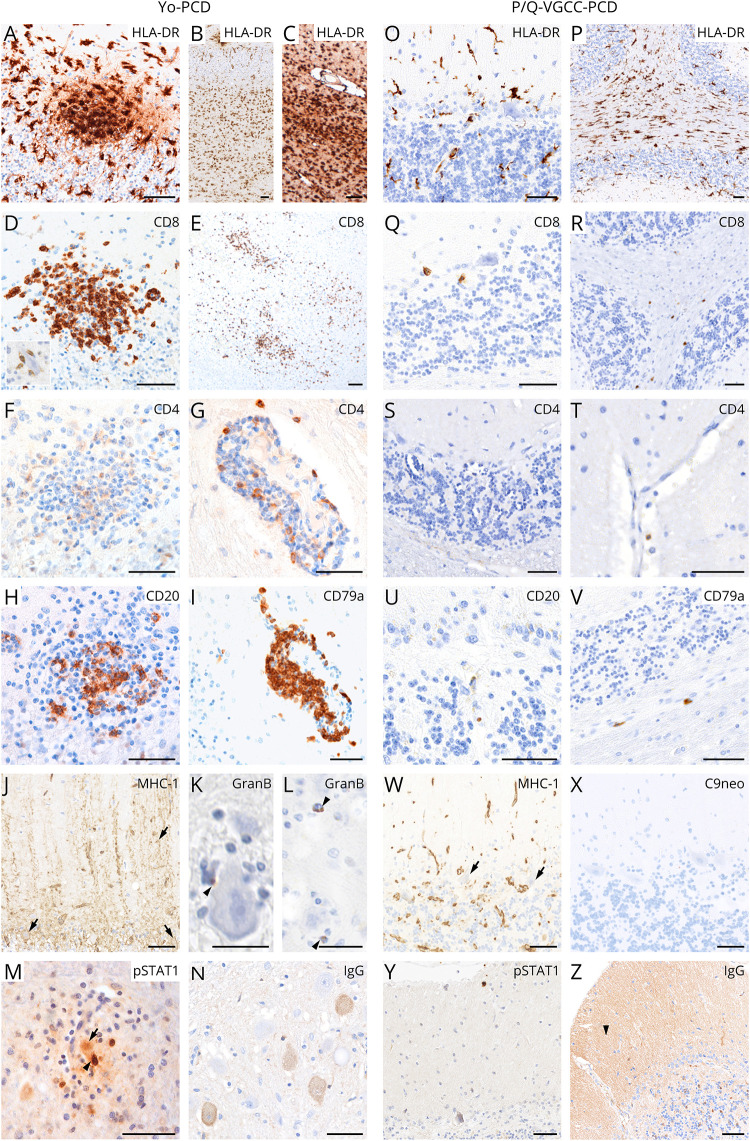
Characterization of Inflammation in PCD Subtypes Inflammation in the cerebellum shows distinct patterns in PCD subtypes. In anti–Yo-PCD, inflammatory nodules were present, comprising predominantly HLA-DR^+^ activated microglia (A–C), cytotoxic CD8^+^granzymeB^+^ T cells (D and E; K and L, arrowheads), single CD4^+^ T cells (F), and CD20^+^ B cells (H). Perivascular cuffs within the cWM were composed of CD4^+^ T cells (G) and mostly CD79a^+^ B/plasma cells (I). Single remaining PCs showed an upregulation of MHC-I molecules (J, arrows). In the dentate nucleus, prominent infiltrates of activated microglia (C), CD8^+^ CTLs (E), and diffuse cytoplasmic IgG deposits in single neurons (N) were shown. In anti–Yo-PCD, pSTAT1 was strongly positive in nuclei of various cells including neurons (arrow) in areas that showed local T-cell attachment (arrowhead) to neurons (M). pSTAT1 was not significantly expressed in anti–P/Q-VGCC-PCD (Y). In anti–P/Q-VGCC-PCD, distribution of HLA-DR^+^ microglia did not present cortical nodules (O) and was accentuated in the cWM (P). CD8^+^ T cells (Q and R) and CD20^+^/CD79a^+^ B/plasma cells (U and V) were scattered mainly within the cWM, accompanied by single perivascular CD4^+^ T cells (S and T). MHC Class I was negative in the remaining PCs (W, arrows) and complement (C9neo) was not detected (X), but IgG immunoreactivity was positive in PCs, Golgi cells, and dentate nucleus neurons (not shown), as well as strongly in the neuropil (Z, arrowhead). Scale bars: 50 μm (except K, L: 25 μm). cWM = cerebellar white matter; HLA = human leukocyte antigen; immunoglobulin G; MHC = major histocompatibility complex; PC = Purkinje cell; PCD = paraneoplastic cerebellar degeneration; P/Q-VGCC = P/Q-type voltage-gated calcium channel.

Inflammation in anti–Yo-PCD was characterized by microglial nodules within the cortical PC layer and dentate nucleus (Figure 4, A–C), cytotoxic CD8^+^granzymeB^+^ T-cell (CTL) infiltrates (Figure 4, D, K and L and 6A, arrowheads mark granzymeB^+^ T cells attached to PCs), and an upregulation of MHC Class I molecules in the remaining PCs (Figure 4J, arrows). Human leukocyte antigen (HLA)-DR was strongly upregulated in activated microglia within the dentate nucleus (Figure 4C), throughout the cerebellar white matter (cWM) and in the cerebellar cortex (Figure 4, A and B). CTLs within the dentate nucleus (Figure 4E) and cWM were accompanied by nodular parenchymal and perivascularly accentuated CD4^+^ T cells (Figure 4, F and G) and CD20^+^/CD79a^+^ B/plasma cells (Figure 4, H and I). In addition, moderate infiltrates consisting of CD3^+^, CD8^+^, CD20^+^, and CD79a^+^ cells were observed in the cerebellar meninges (not shown). pSTAT1, as a marker for interferon (IFN) signaling, was strongly upregulated in microglia and T cells in microglial nodules (Figure 4M, arrowhead, and Figure 6B, arrow) as well as in neurons entrapped in inflammatory nodules in the dentate nucleus (Figure 4M, arrow, and Figure 6C, arrows). Few remaining PCs in the cerebellar cortex that were not surrounded by inflammatory cells were pSTAT1 negative (data not shown). IgG immunolabeling showed diffuse cytoplasmic IgG deposits in single remaining PCs and dentate nucleus neurons, but not in the neuropil (Figure 4N). In contrast, in anti–P/Q-VGCC-PCD, microglial nodules were absent (Figure 4O), and the cWM showed a moderate activation of HLA-DR^+^ microglia (Figure 4P). CD8^+^ T cells were few (Figure 4, Q and R) and accompanied by single parenchymal and perivascular CD4^+^ T cells (Figure 4, S and T) and CD20^+^/CD79a^+^ B/plasma cells (Figure 4, U and V). No neuronal MHC Class I upregulation (Figure 4W, arrows), activated complement complex depositions (C9neo) (Figure 4X), or pSTAT1 expression was found (Figure 4Y). IgG immunolabeling showed a significant neuropil staining in the molecular layer of the cerebellar cortex (Figure 4Z, arrowhead) and some diffuse cytoplasmic staining within PCs, Golgi cells, and neurons of the dentate nucleus (data not shown).

#### Anti–P/Q-VGCC-PCD Shows Reduced Synaptophysin and an Altered Receptor Expression

**Figure 5:**
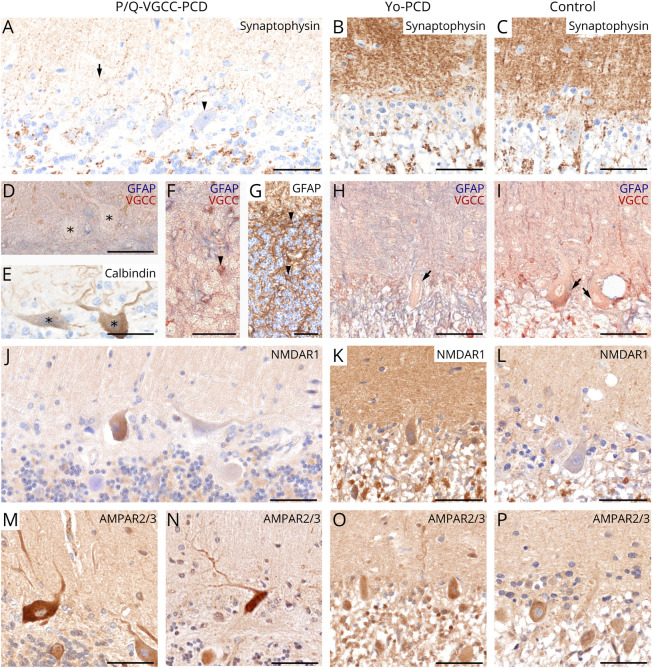
Expression Patterns of Synaptic and Receptor Proteins in PCD Subtypes Composition of synaptic proteins and calcium-related ion channels vary in PCD subtypes. Synaptophysin was strongly reduced within the molecular layer (arrow) and on PCs (arrowhead) in anti–P/Q-VGCC-PCD (A), compared with anti–Yo-PCD and controls (B and C). In anti–P/Q-VGCC-PCD, immunoreactivity of P/Q-VGCC was strongly reduced within the cytoplasm and on dendrites of PCs (D, asterisks), but preserved in anti–Yo-PCD (H, arrow) and controls (I, arrows). Corresponding P/Q-VGCC–negative PCs showed a weak calbindin expression (E, asterisks). Double labeling of GFAP and P/Q-VGCC revealed a strong calcium receptor upregulation on reactive astrocytes (arrowheads) and within Bergmann astrogliosis in anti–P/Q-VGCC-PCD (F and G). Immunohistochemical staining of NMDAR1 appeared to be weakly expressed within the granular cell layer in anti–P/Q-VGCC-PCD (J), compared with anti–Yo-PCD and controls (K and L). AMPAR2/3 was strongly expressed on PCs and its dendrites in anti–P/Q-VGCC-PCD (M and N), in contrast to anti–Yo-PCD and controls (O and P). Scale bars: 50 μm. PC = Purkinje cell; PCD = paraneoplastic cerebellar degeneration; P/Q-VGCC = P/Q-type voltage-gated calcium channel.

**Figure 6:**
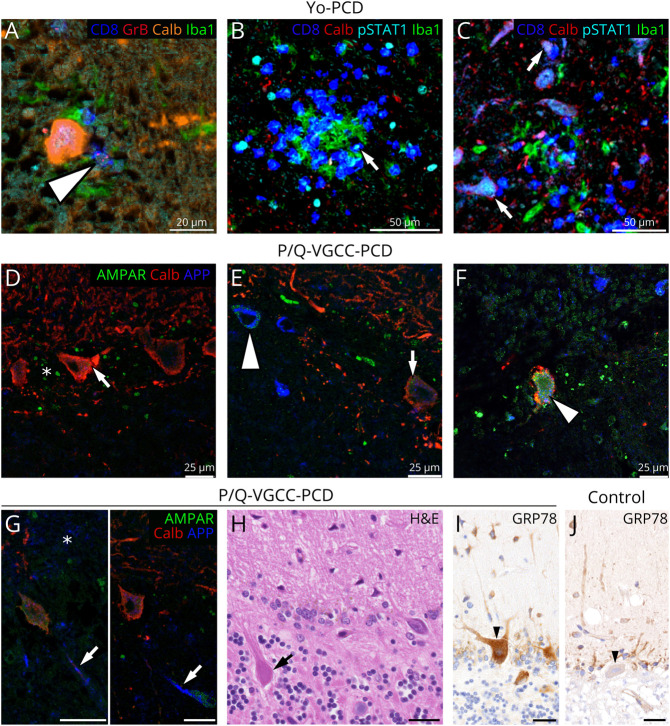
Pathophysiologic Mechanisms in PCD Subtypes Multiplex immunofluorescence in anti–Yo-PCD presents cytotoxic CD8^+^granzymeB^+^ T cells closely attached to PCs (A, arrowhead) and within microglial nodules (B and C). In microglial nodules, pSTAT1 is upregulated in CD8^+^ T cells (B, arrow), Iba1^+^ microglia, and neurons (C, arrows). In areas of anti–P/Q-VGCC-PCD with preserved calbindin expression, a regular AMPAR2/3 expression is present within the molecular layer (asterisk) and in the cytoplasm of PCs (D and E, arrows). Cytoplasmatic AMPAR2/3 expression is enhanced in APP-positive PCs (E, arrowhead) and even stronger expressed in APP-positive PCs in cortical areas with a reduced or lost calbindin reactivity (F, arrowhead). In calbindin-weak or -negative regions (G, asterisk) with severe PC loss, axons show APP accumulation (G, arrows) and axonal spheroids (H, arrow). Single preserved PCs (I, arrowhead) within this region upregulate GRP78 as an endoplasmatic reticulum chaperon involved in unfolded protein response, compared with controls (J, arrowhead). Scale bars: A: 20 μm; B and C: 50 μm, D–J: 25 μm. APP = amyloid precursor protein; GRP78 = glucose-regulated protein 78; PC = Purkinje cell; PCD = paraneoplastic cerebellar degeneration; P/Q-VGCC = P/Q-type voltage-gated calcium channel.

Synaptophysin was strongly reduced in the molecular layer (arrow), on PCs (arrowhead) and its dendrites in anti–P/Q-VGCC-PCD (Figure 5A), compared with anti–Yo-PCD and controls (Figure 5, B and C). The reduced synaptophysin expression was accompanied by a rearrangement of synaptic calcium-related receptor proteins (P/Q-VGCC, NMDAR1, and AMPAR2/3). In anti–P/Q-VGCC-PCD, the expression of P/Q-VGCCs was reduced on dendrites and within the cytoplasm of PCs (Figure 5D, asterisks), compared with anti–Yo-PCD and controls (Figure 5, H and I, arrows). In parallel, respective P/Q-VGCC–negative PCs also showed a reduced calbindin immunoreactivity (Figure 5E, asterisks). In comparison, an overexpression of P/Q-VGCCs was detected on reactive astrocytes within the granular cell layer and in Bergmann glia (Figure 5, F and G, arrowheads). Within the granular cell layer including the glomerula cerebellaria, NMDAR seemed to be reduced in anti–P/Q-VGCC-PCD, compared with anti–Yo-PCD and controls (Figure 5, J–L). AMPAR2/3 immunoreactivity did not appear to be reduced in the molecular layer of anti–P/Q-VGCC-PCD, despite reduced synaptophysin expression (Figures 5, M and N and 6D, asterisk). However, calbindin-negative PCs showed a cytoplasmic accumulation of amyloid precursor protein (APP) (Figure 6E, arrowhead) and AMPAR2/3 protein (Figures 5, M and N and 6F, arrowhead), which was not found in unaffected PCs (Figure 6D and E, arrows), in anti–Yo-PCD and controls (Figure 5, O and P). In anti–P/Q-VGCC-PCD, axons also accumulated APP (Figure 6G, arrows) and formed axonal spheroids (Figure 6H, arrow) in affected regions. As a marker for endoplasmatic reticulum stress and unfolded protein response, preserved PCs strongly expressed glucose-regulated protein 78 (GRP78) (Figure 6I, arrowhead), compared with controls (Figure 6J, arrowhead).

## Discussion

In this study, we compared the neuropathology of anti–Yo- with anti–P/Q-VGCC-PCD. We observed that the neuronal loss as well as type and spatial distribution of inflammation is clearly different between these 2 PCD subtypes, supporting the concept of distinct underlying pathophysiologies.

In our 2 anti–Yo-PCD cases, we observed a generalized loss of PCs that was associated with Bergmann astrogliosis and neuronal cell loss within the dentate nucleus.^1,6^ In comparison, anti–P/Q-VGCC-PCD showed a focal loss of PCs and Golgi cells in the upper vermis. A focal affection of the vermis may also be observed in other PCD subtypes associated with intracellular or surface antibodies and might reflect an increased vulnerability of this region.^2,24^ Of interest, we found a coextensive loss of Golgi cells along with PCs, which may either be a secondary phenomenon after PC loss or might reflect an additional target of the autoimmune reactivity. Because P/Q-VGCCs are not only labeled in the cytoplasm of PCs, but also expressed at synapses of parallel and mossy fibers, both Purkinje and Golgi cells might be functionally impaired by pathogenic antibodies.^25^ Furthermore, we observed that anti–Yo-PCD but not anti–P/Q-VGCC-PCD showed characteristics of a T cell–mediated pathology, including MHC Class I upregulation of PCs as well as microglial nodules with pSTAT1^+^, CD8^+^, and granzymeB^+^ T cells attached to neurons.^1,3,7,9^ Anti–Yo-PCD also showed prominent perivascularly accentuated CD4^+^ T cells and CD20^+^/CD79a^+^ B/plasma cell cuffs, compared with single parenchymal and perivascular CD20^+^/CD79a^+^ B/plasma cells in anti–P/Q-VGCC-PCD. In general, the quantity of inflammatory infiltrates was much less in anti–P/Q-VGCC-PCD. Our patient underwent extensive immunotherapy, which may explain the low amount of inflammation as it was recently also described in 1 treated case of anti-NMDAR encephalitis.^26^

Concepts of T cell–mediated types of encephalitis associated with intracellular antigens like Yo were previously established.^1,3,9,27^ This is in line with our finding in anti–Yo-PCD, where T cells, microglia, and neurons showed a strong pSTAT1 expression. In addition, we detected neuronal MHC Class I upregulation, which most likely represents the result of a complex signal transduction pathway involving the production of IFN-γ by cerebellum-invading T cells and upregulation of pSTAT1. Recently, IFN-γ–induced pSTAT1 signaling was also shown to be essentially involved in creating a proinflammatory milieu and T cell–mediated cytotoxicity in Rasmussen encephalitis.^28,29^ In vitro studies demonstrated an internalization of anti-Yo antibodies in PCs, resulting in neuronal calcium dysregulation, suggesting a pathogenic role of these antibodies, possibly by interfering with the ribosome function due to CDR2L binding.^10^ In our study, we observed a diffuse cytoplasmic IgG staining in some neurons within the dentate nucleus, which may support the in vitro findings of antibody internalization, although a nonspecific IgG uptake due to a leakiness of damaged neurons cannot be excluded.^30^ We also found a diffuse cytoplasmic neuronal IgG staining in anti–P/Q-VGCC-PCD, but additionally observed a strong neuropil staining in the molecular layer of the cerebellar cortex resembling the immunolabeling of P/Q-VGCCs.^18,20^ Because P/Q-VGCCs are highly expressed at synapses within the cerebellar cortex contributing to calcium-dependent exocytosis of neurotransmitters, calcium homeostasis, and synaptic plasticity, pathogenic antibodies may have detrimental effects on neuronal plasticity and survival.^18,25^ Functional blocking may decrease calcium currents and impair cerebellar synaptic transmission via parallel fibers, causing functional impairment and subsequent PC death over time.^15,19,20,31^ Intrathecal injection of a patient's anti–P/Q-VGCC antibodies in mice was shown to cause reversible cerebellar symptoms, indicating antibody pathogenicity. The reversibility, however, might be time dependent.^19,20^ Using autoradiography, the quantity of cerebellar P/Q-VGCCs was previously described to be extensively reduced in 3 autopsies of patients with PCD-LEMS, in particular within the molecular layer.^18^ We confirm immunohistochemically that the expression of P/Q-VGCCs is reduced on dendrites and within the cytoplasm of PCs in anti–P/Q-VGCC-PCD. Moreover, synaptophysin was reduced in respective cortical layers. Within the granular cell layer, double labeling with GFAP revealed an upregulation of the calcium channel on reactive astrocytes, which might reflect a compensatory phenomenon and may aggravate calcium stress and neurodegeneration. We further investigated the synaptic composition of calcium-associated ion channels, demonstrating an overexpression of AMPAR2/3 on PC dendrites and its cytoplasm in calbindin-weak or -negative areas, which is normally inhibited by P/Q-VGCC–mediated mechanisms.^25^ In contrast, NMDAR appeared only weakly expressed on glomerula cerebellaria in anti–P/Q-VGCC-PCD, compared with anti–Yo-PCD and controls. The presence of axonal spheroids within the molecular layer was accompanied by an accumulation of cytoplasmatic proteins (APP, AMPAR2/3, and GRP78) in PCs, indicating endoplasmatic reticulum stress, unfolded protein response, and impairment of axonal transport.^32-34^ Furthermore, the immunoreactivity of calbindin was reduced or lost in the remaining PCs in both PCD types, compared with controls. These observations possibly explain ongoing cellular stress, resulting in PC death caused by altered synaptic transmission and high levels of free intracellular calcium.^35-37^ In anti–P/Q-VGCC-PCD, antibodies may therefore initially cause functional impairment of PCs, followed by an irreversible neuronal damage after longer exposure.^20,31^ Overall, our neuropathologic findings support a pathogenic role of anti–P/Q-VGCC antibodies in neurodegenerative processes of PCD. However, because we do not know whether the neuropathologic changes seen here are also characteristically present in other anti–P/Q-VGCC patients, future collaborative studies with more cases will be necessary to confirm these observations.

The distinct neuropathologic findings of PCD subtypes are very well reflected by the differences in the patients' disease course and therapy response. Clinically, all 3 patients with PCD presented with a rapidly progressive cerebellar syndrome.^4^ Nevertheless, the 2 anti–Yo-PCD patients deceased after relatively short neurologic disease courses of 3 to 5 months. The anti–P/Q-VGCC-PCD patient died after a significantly longer disease duration of 42 months. However, despite the longer disease course, the severity of PC loss was less in our anti–P/Q-VGCC-PCD patient than in our T cell–mediated anti–Yo-PCD cases.^3,6,38^ Compared with the 2 anti–Yo-PCD patients, who did not respond to chemo- or immunosuppressive therapy, the anti–P/Q-VGCC-PCD patient repeatedly improved after oncologic and immunologic therapies. In particular, PLEX therapy and IVIG cycles showed rapid and significant effects, possibly supporting the idea of a pathogenic anti–P/Q-VGCC antibody, which could cause reversible, functional impairment of cerebellar synaptic transmission.^18-20^ In anti–Yo-PCD, a future therapeutic option might possibly be found in IFN-γ–neutralizing antibodies, which reduced cerebellar T-cell infiltration and prevented neuronal destruction in a mouse model of PCD.^39^ Further translational and clinical studies will be necessary to address these relevant questions.

In conclusion, our study reveals important findings: (1) anti–Yo-PCD but not anti–P/Q-VGCC-PCD showed characteristic features of a T cell–mediated pathology; (2) our neuropathologic findings support the concept of a pathogenic role of anti–P/Q-VGCC autoantibodies in PCD in causing neuronal dysfunction, probably due to altered synaptic transmission resulting in calcium dysregulation and subsequent PC death after long-term exposure to the antibodies; and (3) because disease progression may lead to irreversible PC and Golgi cell loss, anti–P/Q-VGCC-PCD patients could benefit from early oncologic and immunologic therapies.

## Glossary

APP: amyloid precursor protein
CTL: cytotoxic T cell
cWM: cerebellar white matter
FFPE: formalin-fixed and paraffin-embedded
GRP78: glucose-regulated protein 78
HLA: human leukocyte antigen
IFN: interferon
IgG: immunoglobulin G
IHC: immunohistochemistry
IVIG: IV immunoglobulin
LEMS: Lambert-Eaton myasthenic syndrome
MHC: major histocompatibility complex
mRS: modified Rankin Scale
PC: Purkinje cell
PCD: paraneoplastic cerebellar degeneration
PLEX: plasma exchange
P/Q-VGCC: P/Q-type voltage-gated calcium channel

## References

[1] Verschuuren J, Chuang L, Rosenblum MK, et al. Inflammatory infiltrates and complete absence of Purkinje cells in anti-Yo-associated paraneoplastic cerebellar degeneration. Acta Neuropathol. 1996;91(5):519-525. 8740233 10.1007/s004010050460

[2] Mason W, Graus F, Lang B, et al. Small-cell lung cancer, paraneoplastic cerebellar degeneration and the Lambert-Eaton myasthenic syndrome. Brain. 1997;120(pt 8):1279-1300. 9278623 10.1093/brain/120.8.1279

[3] Dalmau J, Rosenfeld MR. Paraneoplastic syndromes of the CNS. Lancet Neurol. 2008;7(4):327-340. 18339348 10.1016/S1474-4422(08)70060-7 PMC2367117

[4] Graus F, Vogrig A, Muñiz-Castrillo S, et al. Updated diagnostic criteria for paraneoplastic neurologic syndromes. Neurol Neuroimmunol Neuroinflamm. 2021;8(4):e1014. 34006622 10.1212/NXI.0000000000001014 PMC8237398

[5] Höftberger R, Rosenfeld MR, Dalmau J. Update on neurological paraneoplastic syndromes. Curr Opin Oncol. 2015;27(6):489-495. 26335665 10.1097/CCO.0000000000000222 PMC4640358

[6] Peterson K, Rosenblum MK, Kotanides H, Posner JB. Paraneoplastic cerebellar degeneration: I. A clinical analysis of 55 anti-Yo antibody-positive patients. Neurology. 1992;42(10):1931-1937. 1407575 10.1212/wnl.42.10.1931

[7] Aboul-Enein F, Höftberger R, Buxhofer-Ausch V, et al. Neocortical neurones may be targeted by immune attack in anti-Yo paraneoplastic syndrome. Neuropathol Appl Neurobiol. 2008;34(2):248-252. 17995920 10.1111/j.1365-2990.2007.00909.x

[8] Graus F, Illa I, Agusti M, Ribalta T, Cruz-Sanchez F, Juarez C. Effect of intraventricular injection of an anti-Purkinje cell antibody (anti-Yo) in a Guinea pig model. J Neurol Sci. 1991;106(1):82-87. 1779243 10.1016/0022-510x(91)90198-g

[9] Albert ML, Darnell JC, Bender A, Francisco LM, Bhardwaj N, Darnell RB. Tumor-specific killer cells in paraneoplastic cerebellar degeneration. Nat Med. 1998;4(11):1321-1324. 9809559 10.1038/3315

[10] Schubert M, Panja D, Haugen M, Bramham CR, Vedeler CA. Paraneoplastic CDR2 and CDR2L antibodies affect Purkinje cell calcium homeostasis. Acta Neuropathol. 2014;128(6):835-852. 25341622 10.1007/s00401-014-1351-6 PMC4231287

[11] Greenlee JE, Clawson SA, Hill KE, Wood BL, Tsunoda I, Carlson NG. Purkinje cell death after uptake of anti-Yo antibodies in cerebellar slice cultures. J Neuropathol Exp Neurol. 2010;69(10):997-1007. 20838245 10.1097/NEN.0b013e3181f0c82b PMC2959164

[12] Greenlee JE, Clawson SA, Hill KE, et al. Anti-Yo antibody uptake and interaction with its intracellular target antigen causes Purkinje cell death in rat cerebellar slice cultures: a possible mechanism for paraneoplastic cerebellar degeneration in humans with gynecological or breast cancers. PLoS One. 2015;10(4):e0123446. 25885452 10.1371/journal.pone.0123446 PMC4401511

[13] Graus F, Lang B, Pozo-Rosich P, Saiz A, Casamitjana R, Vincent A. P/Q type calcium-channel antibodies in paraneoplastic cerebellar degeneration with lung cancer. Neurology. 2002;59(5):764-766. 12221175 10.1212/wnl.59.5.764

[14] Sabater L, Höftberger R, Boronat A, Saiz A, Dalmau J, Graus F. Antibody repertoire in paraneoplastic cerebellar degeneration and small cell lung cancer. PLoS One. 2013;8(3):e60438. 23536908 10.1371/journal.pone.0060438 PMC3607586

[15] Liao YJ, Safa P, Chen YR, Sobel RA, Boyden ES, Tsien RW. Anti-Ca2+ channel antibody attenuates Ca2+ currents and mimics cerebellar ataxia in vivo. Proc Natl Acad Sci USA. 2008;105(5):2705-2710. 18272482 10.1073/pnas.0710771105 PMC2268200

[16] Verschuuren JJ, Dalmau J, Tunkel R, et al. Antibodies against the calcium channel beta-subunit in Lambert-Eaton myasthenic syndrome. Neurology. 1998;50(2):475-479. 9484375 10.1212/wnl.50.2.475

[17] Pellkofer H, Armbruster L, Krumbholz M, et al. Lambert–Eaton myasthenic syndrome differential reactivity of tumor versus non-tumor patients to subunits of the voltage-gated calcium channel. J Neuroimmunol. 2008;204(1-2):136-139. 18809213 10.1016/j.jneuroim.2008.08.002

[18] Fukuda T, Motomura M, Nakao Y, et al. Reduction of P/Q-type calcium channels in the postmortem cerebellum of paraneoplastic cerebellar degeneration with Lambert-Eaton myasthenic syndrome. Ann Neurol. 2003;53(1):21-28. 12509844 10.1002/ana.10392

[19] Martín-García E, Mannara F, Gutiérrez-Cuesta J, et al. Intrathecal injection of P/Q type voltage-gated calcium channel antibodies from paraneoplastic cerebellar degeneration cause ataxia in mice. J Neuroimmunol. 2013;261(1-2):53-59. 23726906 10.1016/j.jneuroim.2013.05.003

[20] McKasson M, Clardy SL, Clawson SA, et al. Voltage-gated calcium channel autoimmune cerebellar degeneration: case and study of cytotoxicity. Neurol Neuroimmunol Neuroinflamm. 2016;3(3):e222. 27088118 10.1212/NXI.0000000000000222 PMC4821674

[21] Blumenfeld AM, Recht LD, Chad DA, DeGirolami U, Griffin T, Jaeckle KA. Coexistence of Lambert-Eaton myasthenic syndrome and subacute cerebellar degeneration: differential effects of treatment. Neurology. 1991;41(10):1682-1682. 1922820 10.1212/wnl.41.10.1682

[22] Bauer J, Lassmann H. Neuropathological techniques to investigate central nervous system sections in multiple sclerosis. Methods Mol Biol. 2016;1304:211-229. 25520281 10.1007/7651_2014_151

[23] Motomura M, Johnston I, Lang B, Vincent A, Newsom-Davis J. An improved diagnostic assay for Lambert-Eaton myasthenic syndrome. J Neurol Neurosurg Psychiatry. 1995;58(1):85-87. 7823075 10.1136/jnnp.58.1.85 PMC1073275

[24] Spatola M, Petit Pedrol M, Maudes E, et al. Clinical features, prognostic factors, and antibody effects in anti-mGluR1 encephalitis. Neurology. 2020;95(22):e3012-e3025. 32928978 10.1212/WNL.0000000000010854 PMC7734921

[25] Mitoma H, Honnorat J, Yamaguchi K, Manto M. Fundamental mechanisms of autoantibody-induced impairments on ion channels and synapses in immune-mediated cerebellar ataxias. IJMS. 2020;21(21):4936. 10.3390/ijms21144936 PMC7404345 32668612

[26] Zrzavy T, Endmayr V, Bauer J, et al. Neuropathological variability within a spectrum of NMDAR‐encephalitis. Ann Neurol. 2021;90(5):725-737. 34562035 10.1002/ana.26223

[27] Dalmau J, Graus F. Antibody-mediated encephalitis. N Engl J Med. 2018;378(9):840-851. 29490181 10.1056/NEJMra1708712

[28] Tröscher AR, Wimmer I, Quemada-Garrido L, et al. Microglial nodules provide the environment for pathogenic T cells in human encephalitis. Acta Neuropathol. 2019;137(4):619-635. 30663001 10.1007/s00401-019-01958-5 PMC6426829

[29] Di Liberto G, Pantelyushin S, Kreutzfeldt M, et al. Neurons under T Cell attack coordinate phagocyte-mediated synaptic stripping. Cell. 2018;175(2):458-471.e19. 30173917 10.1016/j.cell.2018.07.049

[30] Bien CG, Vincent A, Barnett MH, et al. Immunopathology of autoantibody-associated encephalitides: clues for pathogenesis. Brain. 2012;135(pt 5):1622-1638. 22539258 10.1093/brain/aws082

[31] Lennon VA, Kryzer TJ, Griesmann GE, et al. Calcium-Channel antibodies in the Lambert–Eaton syndrome and other paraneoplastic syndromes. N Engl J Med. 1995;332(22):1467-1475. 7739683 10.1056/NEJM199506013322203

[32] Bauer J, Bradl M, Klein M, et al. Endoplasmic reticulum stress in PLP-overexpressing transgenic rats: gray matter oligodendrocytes are more vulnerable than white matter oligodendrocytes. J Neuropathol Exp Neurol. 2002;61(1):12-22. 11829340 10.1093/jnen/61.1.12

[33] Hoozemans JJM, Veerhuis R, Van Haastert ES, et al. The unfolded protein response is activated in Alzheimer's disease. Acta Neuropathol. 2005;110(2):165-172. 15973543 10.1007/s00401-005-1038-0

[34] Satoh T, Furuta K, Tomokiyo K, et al. Facilitatory roles of novel compounds designed from cyclopentenone prostaglandins on neurite outgrowth-promoting activities of nerve growth factor. J Neurochem. 2002;75(3):1092-1102. 10.1046/j.1471-4159.2000.0751092.x 10936191

[35] Alexianu ME, Ho BK, Mohamed AH, La Bella V, Smith RG, Appel SH. The role of calcium-binding proteins in selective motoneuron vulnerability in amyotrophic lateral sclerosis. Ann Neurol. 1994;36(6):846-858. 7998770 10.1002/ana.410360608

[36] You JC, Muralidharan K, Park JW, et al. Epigenetic suppression of hippocampal calbindin-D28k by ΔFosB drives seizure-related cognitive deficits. Nat Med. 2017;23(11):1377-1383. 29035369 10.1038/nm.4413 PMC5747956

[37] Westerink RHS, Beekwilder JP, Wadman WJ. Differential alterations of synaptic plasticity in dentate gyrus and CA1 hippocampal area of Calbindin-D28K knockout mice. Brain Res. 2012;1450:1-10. 22405690 10.1016/j.brainres.2012.02.036

[38] Giometto B, Marchiori GC, Nicolao P, et al. Sub‐acute cerebellar degeneration with anti‐Yo autoantibodies: immunohistochemical analysis of the immune reaction in the central nervous system. Neuropathol Appl Neurobiol. 1997;23(6):468-474. 9460712 10.1111/j.1365-2990.1997.tb01323.x

[39] Yshii L, Pignolet B, Mauré E, et al. IFN-γ is a therapeutic target in paraneoplastic cerebellar degeneration. JCI Insight. 2019;4(7):e127001. 10.1172/jci.insight.127001 PMC6483638 30944244

